# From Anatomy to Innovation: Embedding Patient-Centered Design in Medical Education

**DOI:** 10.2196/86230

**Published:** 2026-04-20

**Authors:** Louis John Koizia, Pieter Vandekerckhove, Steven Howard, Bettina Maisch, Benjamin Howell Lole Harris

**Affiliations:** 1 Cutrale Perioperative and Ageing Group, Imperial College London, Sir Michael Uren Hub, 86 Wood Lane, White City Campus, London, W12 7TA, United Kingdom, 44 7765020719; 2 Delft Centre for Entrepreneurship, Delft University of Technology, Delft, The Netherlands; 3 Department of Health Services Administration, School of Health Professions, University of Alabama at Birmingham, Birmingham, AL, United States; 4 Department of Applied Sciences and Mechatronics, Munich University of Applied Sciences, Munich, Germany; 5 Department of Oncology, University of Oxford, Oxford, United Kingdom

**Keywords:** educational innovation, value-based health care, reform, curriculum, transformative learning

## Abstract

Medical education has traditionally focused on mastering biomedical knowledge, clinical skills, and professionalism. Yet it remains poorly equipped to prepare doctors for the complex, rapidly changing health systems they will work in. Despite repeated calls for transformative learning, progress in teaching system innovation has been limited. Current curricula produce clinicians adept at treating disease but often underprepared to address system failures or contribute to organizational change. Embedding patient-centered innovation within training may help address this gap. This approach integrates design thinking, value-based health care, and co-design with patients to cultivate the metacompetence required to engage with system change. Emerging examples suggest that innovation education is most effective when integrated within existing activities such as quality improvement initiatives, multidisciplinary collaboration, and protected time for improvement work. Teaching these principles early may help normalize innovation as an integral component of clinical practice rather than a peripheral activity. Lessons from the successful integration of communication skills and ethics into medical curricula demonstrate that such cultural shifts are possible. Practical approaches may include incorporating design-thinking exercises, patient co-creation activities, and innovation projects alongside existing quality improvement and leadership programs. Reframing innovation as part of professional responsibility may empower clinicians to act as catalysts for system improvement. From anatomy to innovation, medical education must continue to evolve, preparing doctors not only to treat patients but also to engage with the systems that shape health care delivery.

## Introduction

Medical education has traditionally focused on equipping students with a strong foundation in biological sciences, clinical skills, and professional values essential for safe and compassionate care [1]. Curricula emphasize anatomy, physiology, pathology, and pharmacology, alongside integrative competencies such as communication, ethics, and teamwork. Within medical education scholarship, these competencies are commonly conceptualized through competency-based medical education frameworks and theories of experiential learning, which emphasize the progressive development of knowledge, skills, and professional identity through practice and reflection. However, a persistent mismatch remains between the competencies taught and the evolving needs of health systems.

In response, many programs have introduced transformative learning elements, including leadership and digital health. Yet, from the student perspective, curricula still lack sufficient emphasis on digital health and practical experience with emerging technologies [1-4]. Despite improvements, the gaps identified by Frenk et al [1] in 2010 persist. Future clinicians are not adequately prepared to address systemic challenges such as aging populations, multimorbidity, digital disruption, and the demand for value-based care.

Graduates are trained to diagnose and treat disease but often lack the skills to identify and address system-level failures. Without the skills to innovate, clinicians are sidelined in system transformation efforts, which are typically led by policymakers and managers. We argue for a renewed focus on competencies that empower clinicians to drive health system innovation and enhance patient-centered care.

**Figure 1.**
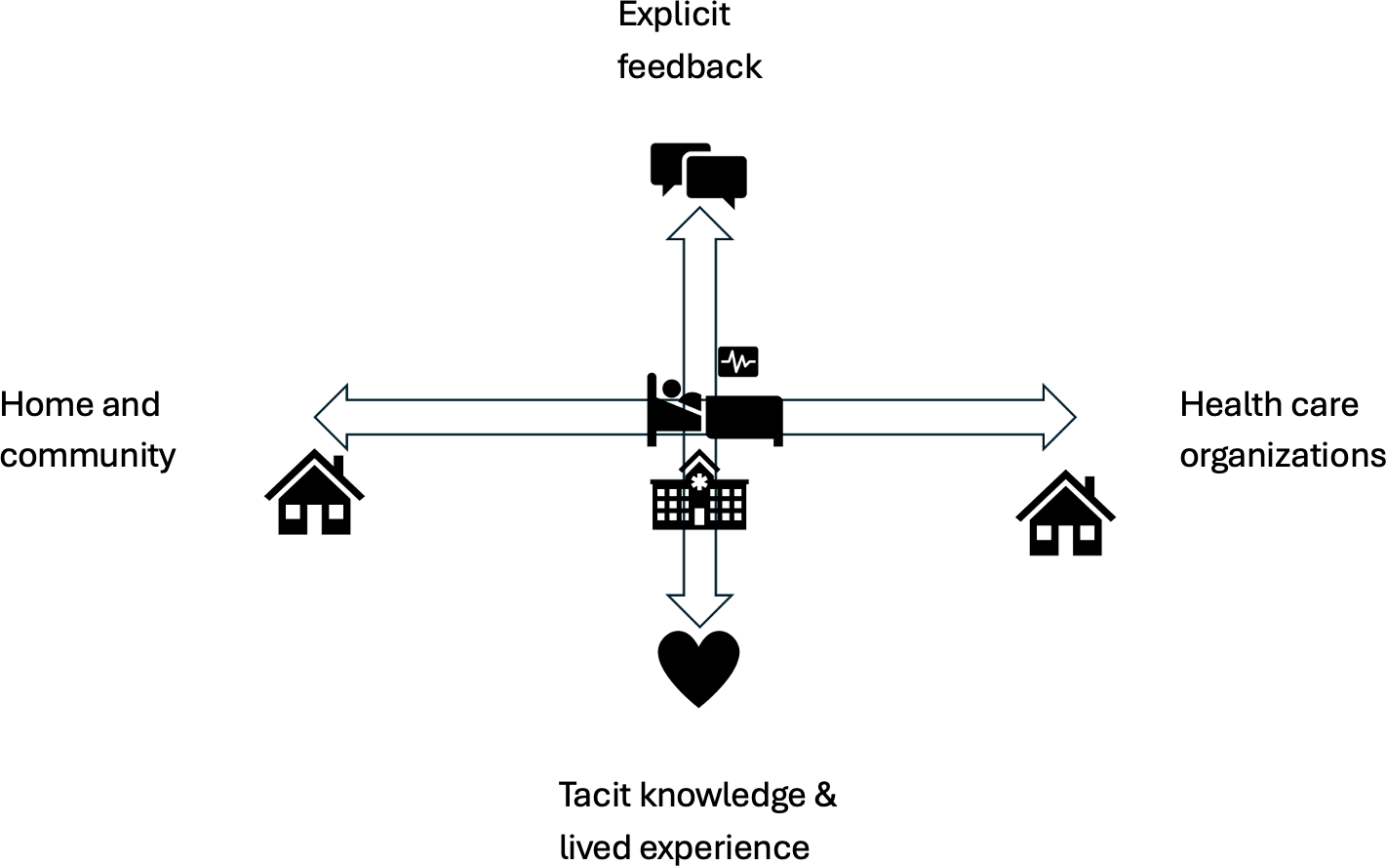
Conceptual framework for patient-centered innovation in medical education*.* The vertical dimension represents the depth of stakeholder understanding, ranging from explicit feedback to deeper emotional and tacit knowledge uncovered through participatory design approaches. The horizontal dimension represents the integration of innovations across the patient journey, linking home environments, community services, and health care organizations within the broader health care value chain*.*

In this article, we propose a conceptual framework for integrating patient-centered innovation into medical education. The framework distinguishes between 2 complementary dimensions of system improvement: vertical integration, which focuses on deep engagement with stakeholder experience and tacit knowledge, and horizontal integration, which focuses on positioning innovations across the broader patient journey and health care value chain. By articulating these dimensions, the framework aims to provide a conceptual structure for understanding how innovation competencies may be integrated into medical education and applied within complex health care systems (Figure 1).

## The Systemic Gap in Medical Training

Many medical schools remain anchored to a 20th-century model centered on biomedical knowledge, clinical competence, and professional ethics. While these pillars are essential, they are insufficient in today’s complex health care landscape. Scholars in health professions education increasingly highlight the need for clinicians to develop capabilities that extend beyond individual patient encounters toward system awareness, interdisciplinary collaboration, and adaptive problem solving. Importantly, not all medical students aspire to traditional clinical roles. A recent Finnish survey revealed that approximately one-third of medical students consider product development a career path [5].

Health care systems face workforce shortages, widening health inequities, and rapid technological change. To navigate this disruption, students need metacompetence, defined as the ability to develop new competencies in response to evolving challenges and to adapt knowledge and skills across changing professional contexts [5,6]. This includes understanding how to lead change across organizational and technological boundaries and how to collaborate across disciplines. Students must understand the competencies required to implement change successfully and how innovations become embedded within complex interactions between people, organizations, and technologies.

This metacompetence requires the ability to apply skills from entrepreneurship, innovation, leadership, technology, and human-centered design. While some programs have begun to introduce these areas, they often lack integration [7]. Globally, health entrepreneurship and innovation education focused on addressing complex system challenges remain underdeveloped and universities struggle to embed it into medical curricula [8].

## Conceptual Framework for Patient-Centered Innovation in Medical Education

To improve care at a systemic level, metacompetence must be cultivated. The conceptual framework proposed in this paper (Figure 1) illustrates how innovation competencies can be understood through 2 interacting dimensions: depth of stakeholder understanding (vertical integration) and alignment across health care pathways and organizations (horizontal integration).

This perspective aligns with conceptualizations of health care as a complex adaptive system, in which outcomes emerge from interactions between individuals, organizations, and technologies rather than from isolated interventions. Within such systems, clinicians require capabilities that extend beyond individual clinical decision-making toward understanding relationships, feedback loops and system dynamics. The proposed framework therefore situates innovation competencies within the broader context of system-aware clinical practice.

Patient-centered innovation requires skills in design thinking, stakeholder co-creation, and value-based health care [9]. In this article, patient-centered innovation refers to the development and implementation of health care solutions that are co-designed with stakeholders and aligned with patient needs, experiences, and outcomes across the care pathway. These intersecting disciplines must be introduced in ways that highlight their relevance to clinical practice. Medical students should therefore learn to communicate and collaborate with engineers, managers, financial experts, and data scientists.

Educational theory provides useful insights into how such competencies may be developed. Experiential and design-based learning approaches emphasize iterative cycles of problem identification, experimentation, and reflection, making them particularly suited to innovation education within complex health care environments.

Increasingly, health care innovation is driven by advances in genomics, data science, and precision medicine, which are reshaping how diseases are understood and managed. Studies linking genetic variants to key disease phenotypes illustrate the growing importance of integrating large-scale biological data into clinical reasoning and innovation in care delivery [10-12].

This also demands a shift in mindset toward entrepreneurship, experimentation, and collaboration. These developments raise an important question for medical education: whether and how innovation-related competencies should be incorporated into clinical training.

## Why Innovation Belongs in the Curriculum

Innovation in health care is frequently equated with technology. However, true patient-centered innovation aligns system outcomes with what matters most to patients. Several frameworks can guide this integration:

- Design thinking, which is an iterative approach to problem-solving [13]
- Value-based health care, which focuses on patient value rather than service volume [14]
- Experience-based and generative co-design, which positions diverse stakeholders, including care professionals and patients as active partners in service development [9]

Building on these perspectives, the proposed framework emphasizes 2 complementary dimensions: vertical integration of stakeholder insight and horizontal integration across the health care value chain (Figure 1) [15].

## Clinicians as Catalysts for Change

Clinicians are not merely stakeholders in innovation, they are potential catalysts. They shape patient experiences, influence adoption patterns, and guide resource use. Clinicians in leadership roles act as gatekeepers, yet face barriers such as time constraints, hierarchical cultures, misaligned incentives, and fear of failure.

Importantly, clinicians can play a transformative role in design-led innovation, particularly through participatory design and generative co-design approaches [16]. These methods build on design thinking principles and emphasize deep collaboration with stakeholders, including patients, carers, and frontline staff. Participatory design involves users in the early stages of problem definition and solution development, while generative co-design enables stakeholders to articulate latent needs and envision future possibilities through creative exploration.

Understanding patient perspectives is essential in such processes. Research exploring public perceptions demonstrates how attitudes toward emerging health technologies vary across populations [17] and highlights the importance of engaging diverse communities in the development of health care innovations.

Although clinicians are often involved in such projects, they are rarely educated about these methodologies during their training. This lack of exposure limits their ability to lead or fully engage in innovation processes. Embedding education on participatory and generative co-design into medical curricula would empower clinicians to become proactive agents of change, capable of driving systemic improvements from within.

Unless curricula explicitly prepare students to recognize opportunities and navigate these challenges, innovation will remain peripheral. Embedding innovation within quality improvement, patient co-design, and multidisciplinary collaboration is essential. In addition, ring-fencing protected time for engagement can normalize innovation as a core clinical responsibility.

## Lessons From History and Practice

Medical education has successfully integrated new domains before. Communication, once considered peripheral, is now embedded across curricula and assessed in exams. Assessment methods have also evolved, with structured question formats increasingly used to evaluate the application of clinical knowledge in modern medical training [18]. Similarly, medical ethics, professionalism, and interprofessional teamwork have moved from the margins to the mainstream [19]. These shifts reflect a growing recognition that excellence in medicine requires more than scientific knowledge.

However, medical education must also be prepared to change gears, even when the broader health system evolves slowly. Curricula must equip learners not only to adapt to innovation but to lead it. Increasingly, programs are incorporating health and technology perspectives. For example, Finland has made notable progress in digital health education, as illustrated by a recent study tracking medical student competencies and attitudes between 2016 and 2022 [5,19]. In the Netherlands, Erasmus Medical Centre’s *Arts 2030* initiative focuses more on interprofessional skills and the integration of technology.

Despite these advances, attention to patient-centered innovation within medical education remains limited. While some US programs have introduced entrepreneurship and innovation into medical training, these efforts often fall short of teaching students how to integrate technology in a human-centered way that truly benefits patients across horizontal and vertical dimensions.

To truly prepare future clinicians, medical education must go beyond technical literacy and foster the ability to co-create solutions with patients, embed value-driven principles, and lead systemic change.

## Practical Steps for Integration

Embedding patient-centered innovation need not displace core sciences but should complement them. Rather than introducing entirely new curricular content, innovation competencies may be integrated within existing educational activities such as quality improvement projects, interdisciplinary learning, and experiential problem-solving exercises. For example, students could apply design thinking principles within existing quality improvement modules, use the horizontal and vertical pathway framework when analyzing clinical cases, or examine patient care pathways as part of health care delivery teaching. Clinical placements could also incorporate reflective exercises in which students identify system-level barriers to care and propose potential improvements.

On a policy level, greater attention to patient-centered innovation by medical colleges, professional associations, and accreditation bodies could facilitate the integration of these competencies into training programs. Practical approaches may include:

- Integration of design thinking and value-based care principles into existing case-based teaching
- Patient co-creation exercises incorporated into curriculum design or service improvement projects
- Innovation projects aligned with research or quality improvement initiatives addressing real health care system challenges
- Postgraduate reinforcement through leadership training, quality improvement programs, and specialist education

Global perspectives should also be considered when implementing innovation education. Health care systems differ widely in resources, workforce structures, and educational priorities. In low- and middle-income countries, resource constraints may limit access to digital technologies or dedicated innovation programs, but locally driven problem-solving and frugal innovation may offer valuable educational opportunities. Cultural contexts may also influence how clinicians, patients, and communities engage in co-design processes. Educational approaches therefore need to remain adaptable, allowing institutions to integrate innovation competencies in ways that reflect local health care delivery models, cultural expectations, and available resources. Implementation will also require attention to practical considerations such as faculty development, alignment with accreditation standards, and the availability of institutional resources. Recognizing these factors may help support integration within existing curricula and mitigate resistance from traditional medical education stakeholders.

Importantly, the aim of such training is not to transform all physicians into entrepreneurs or technology developers. Rather, it is to cultivate innovation literacy: the ability to understand system challenges, collaborate across disciplines, and contribute meaningfully to the development and implementation of patient-centered solutions.

While empirical evidence linking innovation education directly to patient outcomes remains limited, emerging examples suggest potential benefits. Several institutions have introduced innovation and design-oriented programs within medical education, including hackathons, interdisciplinary design courses, and health innovation tracks, which have been associated with increased student engagement in quality improvement, digital health, and health system innovation activities. However, robust evaluation frameworks are still developing. Future research should focus on measurable outcomes such as students’ ability to analyze care pathways, collaborate across disciplines, and implement system improvements. Longitudinal studies following graduates exposed to innovation-oriented curricula, as well as comparative studies between traditional and innovation-enhanced training programs, will be important to better understand their impact on health care delivery and patient outcomes.

## Conclusion

Health systems face an uncertain future. Aging populations and chronic disease are intensifying pressures. Clinicians must be equipped not only to treat disease but also to redesign systems of care. This article proposes a conceptual framework for patient-centered innovation education and highlights practical ways such competencies may be integrated into existing medical curricula through quality improvement activities, interdisciplinary learning, and clinical reflection. Embedding these competencies into curricula will not turn every doctor into an entrepreneur, but it may produce clinicians who are better equipped to engage with system improvement and multidisciplinary innovation.

From anatomy to innovation, medical education must evolve. Integrating patient-centered design and value-driven care into undergraduate and postgraduate training may represent an important step toward preparing clinicians to engage with system-level challenges in modern health care.
